# 3D Cell Culture Systems: Tumor Application, Advantages, and Disadvantages

**DOI:** 10.3390/ijms222212200

**Published:** 2021-11-11

**Authors:** Ola Habanjar, Mona Diab-Assaf, Florence Caldefie-Chezet, Laetitia Delort

**Affiliations:** 1 Université Clermont-Auvergne, INRAE, UNH, Unité de Nutrition Humaine, CRNH-Auvergne, 63000 Clermont-Ferrand, France; ola.habanjar@etu.uca.fr (O.H.); florence.caldefie-chezet@uca.fr (F.C.-C.); 2 Equipe Tumorigénèse Pharmacologie Moléculaire et Anticancéreuse, Faculté des Sciences II, Université Libanaise Fanar, Beyrouth 1500, Liban; mdiabassaf@ul.edu.lb

**Keywords:** three-dimensional (3D) culture model, extracellular matrix, hydrogel, tissue engineering, spheroids

## Abstract

The traditional two-dimensional (2D) in vitro cell culture system (on a flat support) has long been used in cancer research. However, this system cannot be fully translated into clinical trials to ideally represent physiological conditions. This culture cannot mimic the natural tumor microenvironment due to the lack of cellular communication (cell-cell) and interaction (cell-cell and cell-matrix). To overcome these limitations, three-dimensional (3D) culture systems are increasingly developed in research and have become essential for tumor research, tissue engineering, and basic biology research. 3D culture has received much attention in the field of biomedicine due to its ability to mimic tissue structure and function. The 3D matrix presents a highly dynamic framework where its components are deposited, degraded, or modified to delineate functions and provide a platform where cells attach to perform their specific functions, including adhesion, proliferation, communication, and apoptosis. So far, various types of models belong to this culture: either the culture based on natural or synthetic adherent matrices used to design 3D scaffolds as biomaterials to form a 3D matrix or based on non-adherent and/or matrix-free matrices to form the spheroids. In this review, we first summarize a comparison between 2D and 3D cultures. Then, we focus on the different components of the natural extracellular matrix that can be used as supports in 3D culture. Then we detail different types of natural supports such as matrigel, hydrogels, hard supports, and different synthetic strategies of 3D matrices such as lyophilization, electrospiding, stereolithography, microfluid by citing the advantages and disadvantages of each of them. Finally, we summarize the different methods of generating normal and tumor spheroids, citing their respective advantages and disadvantages in order to obtain an ideal 3D model (matrix) that retains the following characteristics: better biocompatibility, good mechanical properties corresponding to the tumor tissue, degradability, controllable microstructure and chemical components like the tumor tissue, favorable nutrient exchange and easy separation of the cells from the matrix.

## 1. Introduction

Cell culture systems, applied in biology, have contributed to reducing laboratory animal use and ensured the progression of research, pharmaceutical discovery, and the evolution of medicine [1]. Initially, cells were grown in two dimensions and attached to polystyrene utensils or flat adherent surfaces (2D) but then researchers started to grow them with attachment proteins in a synthesized extracellular matrix (ECM) (3D) [2]. The two-dimensional (2D) in vitro cell culture system is a traditional application on flat support [3,4] for cell growth in a monolayer. Historically, this system has been applied in research since the early 1900s [5,6], specifically in the co-culturing of cellular heterogeneity [7,8], and in oncological research as a tool to evaluate the biological performance of bioactive molecules [7]. On the other hand, this type of culture has many limitations since it cannot ideally mimic physiological conditions and the natural microenvironment such as structure, physiology, biological signals of living tissues, and cell-matrix interactions [9,10,11,12,13,14]. Indeed, the communication of cells with their ECM, which is absent in 2D, controls the cell growth, proliferation, and function [15,16]. Cells cultured in 2D have been forced to modify various complex biological functions such as cell invasion, apoptosis, transcriptional regulation and receptor expression [2,14,17], cell proliferation and anti-apoptosis [18,19]. To overcome these limitations, researchers are currently providing and developing new in vitro 3D cell culture systems to boost research (healthy and tumor) [20,21]. The first 3D cell culture model was provided in 1992 by Petersen and Bissell, who described 3D organotypic structures to mimic breast structures in cancerous and non-cancerous cases [22]. This 3D system mimics the natural physiological properties and conditions, as well as enhances the development of new treatments at the preclinical stage in the future [23,24,25,26]. It overcomes the problems associated with traditional 2D in vitro culture, provides more valuable information about 3D cell-cell and cell-matrix interactions and presents a more clinically representative response to therapeutic agents [24,26,27]. Moreover, such a new type of culture will progressively evolve to have broader objectives to better assess the biological and molecular pathways during malignant transformation [28,29,30]. The main role of 3D culture is therefore to imitate the structure of the ECM of the tissue. ECM is a scaffolding of non-cellular fibrillary proteins, various structural macromolecules (accessory proteins), and adhesive molecules that provide structural and biochemical support to cells and are essential to many basic processes [31,32,33,34,35]. In addition, it forms cell-binding sites that control cell adhesion and migration [36]. From a structural point of view, it is composed of:

(i) Interstitial ECM (stromal) contains biomolecules that can be organized into two main classes (proteins, glycoproteins) and proteoglycans (polysaccharides) [37]. It consists mainly of several protein molecules such as collagen I and III, self-arranged polysaccharides in fiber networks of glycosaminoglycans (GAG) such as hyaluronic acid (HA), proteoglycan (PG) and fibronectin [33,34,38,39,40];
(ii) Basement membrane located at the basal side of epithelial or endothelial cells in normal tissues providing a physical barrier between epithelial cells and connective tissue (stroma) of the organ (always allow gas diffusion and transport of signaling molecules) [37,41,42].

These characteristics and ECM composition can be reached by 3D culture, and 3D culture can be achieved either via scaffold-free structures (i.e., spheroids) or scaffold-based structures (i.e., hydrogel-based supports and hard polymer material-based supports). Thus, in this review, we will be explaining the different techniques to reach 3D culture system, their advantages, and disadvantages.

## 2. 2D versus 3D Cell Culture

Cell-cell and cell-matrix interactions cannot be studied in 2D models in contrast to 3D models that are able to mimic these conditions in vitro. So, 3D culture provides a pragmatic pathophysiological microenvironment [11,13,26] and plays a potential role in cancer drug discovery due to the lack of preclinical models relevant to 2D cultures [25,43,44,45]. Inserts can be made up of biomaterials with properties like the ECM, and cells can also produce certain ECM proteins like collagen. Differences in physical and physiological properties between 2D and 3D cultures make 2D cells more sensitive to the effects of drugs than 3D cells since 2D cells are unable to maintain normal morphology as 3D cells can, and due to the difference in the organization of surface receptors on the cell [46,47]. It should be noted that there is growing evidence suggesting that cells cultured in a 3D system behave differently from those cultured in a 2D system and retain important signals from the ECM [28,48,49,50,51,52,53,54,55]. Appropriate 3D culture thus provides a more physiologically relevant approach for the analysis of gene function and cell phenotype ex vivo [56]. In recent years, reconstructed *3D* culture has become a method of choice for summarizing the tissue architecture of benign and malignant tumors [49,57]. Thus, 3D culture can provide an important tool for better understanding changes, interactions, and cellular and molecular signaling during malignant transformation [13,58,59]. Table 1 summarizes the differences between the characteristics of 2D and 3D culture models.

## 3. Extracellular Matrix Composition

The ECM forms the non-cellular physical support for the cellular constituents of all tissues and organs. The components of the ECM encompass cellular and biomechanical signals that maintain morphogenesis, differentiation, tissue homeostasis, integrity, and elasticity [60,61].

The ECM is divided into two main parts: the structural interstitial matrix surrounding the cells (collagen I and fibronectin) and the basement membrane (collagen IV, VIII and X, laminins, nidogen, perlecan, and integrin receptors) separating the epithelium from the surrounding stroma [62,63,64,65,66]. In mammals, ECM contains about 300 proteins (central matrisome), having 43 collagen subunits, 36 proteoglycans (PG), and about 200 complex glycoproteins [67]. The PG glycosaminoglycan (GAG) (proteoglycans that form the intercellular interstitial gel) [67,68,69] and fibrous proteins (collagen, elastin, fibronectins, laminins) form the essential macromolecules of ECM [60,66,70]. The shape and structure of PGs vary according to their functions in ECM. Three parameters allow the classification of PGs: central proteins, their localization, and their GAG composition (unbranched polysaccharide chains, sulfated or not sulfated) [69]. Normal glandular epithelial tissues, including the breast, are composed of a simple layer of epithelial cells that cover the internal cavity of the canal, their apical pole that is in contact with the light-filled with liquid, and their basal pole that rests on the basement membrane. Then, at the limit with the interstitial ECM (stroma), the layer of myoepithelial basal cells rests [71]. Then the homeostasis of this epithelial tissue depends on communication and reciprocal interactions with the stromal microenvironment [72]. Moreover, ECM is a rich reservoir of growth factors and other bioactive molecules (metabolic precursors). It is due to the signaling of this reserve that, on the one hand, cell proliferation, cell differentiation, and delay of apoptosis take place [73], and on the other hand, the reciprocal interaction of cells and the microenvironment is possible [74,75,76]. In addition, ECM plays a basic role in the development and maintenance of epithelial tissues. For example, the ECM of human breast tissue is composed of protein fibril complexes intertwined in a network of carbohydrate chains of GAGs.

From a structural point of view, protein components, including laminins, fibronectin, and collagens, resist tensile forces, while carbohydrates, composed mainly of hyaluronan chains, chelate water, and resist compressive forces. Thus, the ECM is a key regulator of normal homeostasis and tissue phenotype [77].

Collagen is the most abundant structural protein in human tissues and constitutes about 30% of the body’s total protein mass [42,66]. Generally, collagens, formed of 28 unique subtypes discovered [63,78,79], can be grouped either in fibrillar collagen (collagens I-III, V, and XI) or non-fibrillary [67]. They regulate adhesion, cell migration [42], and tensile strength to maintain homeostasis [80]. Basically, interstitial collagen is secreted by fibroblasts, being able by this to organize the alignment of collagen fibrils (I and III) [81] in the fibronectin, hyaluronic acid, metalloproteinases (MMP), growth factors promoting cell differentiation, growth, and migration [79,82,83].

Hyaluronic acid (HA) is a non-sulfated linear GAG polysaccharide [69] with hydrophilic characteristics and is resistant to high compressive forces. HA adopts very extensive conformations for the formation of hydrogels [60,84]. It is a natural component essential for ECM to maintain compliance with compression and ensure ideal homeostasis in combination with collagen [80]. In addition, its abnormal accumulation in ECM may promote tumor migration [85,86].

Fibronectin is a multidomain protein that interacts with different components of ECM to facilitate cell-ECM connection [66], thus forming a fibrillary network [87]. It is involved first in guiding the direction of the organization of interstitial ECM through its reciprocal assembly with type I collagen [87,88,89,90,91] and second in cell migration during development and tumor metastases [72].

Laminins are glycoproteins involved in adhesion, differentiation, migration, phenotype maintenance, and have resistance to apoptosis [92]. It ensures the assembly of the basement membrane as well as ECM-cell interactions [93,94,95,96].

## 4. Three-Dimensional Cell Culture Scales

Various methods have been developed to address the growing demand for cell culture due to the lack of a single technology that fulfills the needs of all 3D cell cultures. 2D culture omits the effect of ECM molecules. Yet, its density and packaging contribute significantly to the creation of a 3D atmosphere. The 3D model is an in vitro reconstitution of the ECM after being inspired by the native microenvironment. It keeps the geometric, mechanical, and biochemical properties of the ECM [97]. It consists of different cells integrated into a specialized environment arranged in a way that forms 3D tissues similar to the natural tissue structure [98,99]. 3D culture models then make it possible to study the morphology and cellular organization shaped by ECM interactions, which are altered during oncogenic transformation. This makes the 3D models of in vitro tumors essential tools to study the mechanisms of cancer growth and metastasis [55,100,101]. They are most useful when they support tissue growth from primary human cells and include defined and physiologically relevant components [49,57,102,103]. Appropriate 3D culture thus provides a more physiologically relevant approach for the analysis of gene function and cell phenotype ex vivo [56]. The engineering of 3D culture is based on different main principles: the nature of the cells (the selected, isolated, appropriate strain cell line, primary cells, and tissue origin), 3D artificial microenvironment (ECM imitation) in which they are grown, scaffold-based of biomaterials (natural, synthetic, or hard), signaling molecules (proteins and growth factors) and bioreactors (cell culture) that support a cellular environment that is biologically active [104,105,106]. For these reasons, these parameters must be evaluated before choosing the most relevant technique and appropriate model. Culture systems can be either scaffold-based on natural or artificial solid scaffolding or scaffold-free, such as spheroids (non-scaffold based) [104]. The details of scaffold-based 3D techniques overview with these attributes are described in Table 2.

### 4.1. 3D Scaffolding Structures

In these techniques, the cells are grown in the presence of support which can be hydrogel-based supports and hard polymer material-based supports, natural or synthetic, of animal or vegetable origin. The polymer scaffolding offers a three-dimensionality favorable to cellular behavior in the microenvironment [124,125]. It is the most used model, especially collagen-based hydrogel, due to the major constituent elements of the basement membrane. Hydrogels are cross-linked networks formed of hydrophilic polymers attached through physical, ionic, or covalent interactions [126]. Their hydrophilic character allows them to absorb water that penetrates successively between the polymeric chains and causes swelling and thus the formation of the hydrogel [127]. Hydrogels can be natural (natural polymers), synthetic (synthetic polymers) or hybrid (natural and synthetic) depending on the biocompatibility advantage or the physico-chemical nature, respectively [128,129] (Table 2). These polymer-modified structures can be used as a matrix for cell culture in vitro or to make 3D spheroids [16]. The main advantage of hydrogels is that their physico-chemical properties are adjustable and could appropriately mimic the biochemical and mechanical properties of the true native ECM. Cells can be deeply seeded into a porous hydrogel and easily recapitulate nutrition and oxygen (by diffusion) [127].

#### 4.1.1. Hydrogels

Some used hydrogels such as those of collagen are expensive, present a lack of reproducibility, and require extensive handling and specific equipment but present the opportunity of cellular heterogeneity as well as spontaneous cell organization (can be heterogeneous) [8,130,131] (Table 2). Another option is to work with other scaffolding products, such as Hydrogel, which is a synthetic nanofiber peptide scaffolding. The stiffness of the 3D culture can be controlled by adjusting the hydrogel concentration. Above all, this system could be applied to study the interaction between any type of neoplastic cells. It may even be possible to design more complex systems using more than two different cell types [130,132]. Hydrogels are unique because of their ability to mimic ECM while allowing soluble factors such as cytokines and growth factors to travel through tissue-like gel [47]. There are different types of hydrogels: natural and synthetic. Natural gels (natural polymers) are, for example, fibrinogen, HA, collagen, Matrigel, gelatin, chitosan, and alginate [133,134,135]. The hydrogels are, by definition, networks composed of hydrophilic polymers that are not cross-linked, and this allows them to swell widely by covalent bonds or to be held together by intramolecular and intermolecular physical attractions [106,136,137], maintaining their 3D structure [138]. Due to their hydrophilic and hydrated character, they can absorb a large amount (thousands of percent) of water or biological fluids and inflater easily without dissolving, thus mimicking the structures and physical properties of soft tissue ECM. They are then soft and rubbery after swelling and resembling living tissues [106]. On the other hand, gels differ from hydrogels since they are semi-solid materials (can appear more solid than liquid) consisting of hydrophilic polymers comprising small amounts of solids, dispersed in relatively large quantities of liquid [139]. Depending on the nature of the polymer, hydrogels can be classified into different natural or synthetic categories and interconnected by physical and ionic interactions and even covalent bonds (hydrogels based on ECM proteins, natural hydrogels, and synthetic hydrogels) with distinct biochemical, physical and mechanical properties [111,112,113,114,115,138]. For this, they were also explored as 3D models for cancer research. The use of 3D scaffolding models based on scaffolding expands the range of options available to researchers [140,141,142,143,144,145,146,147,148].

- Protein-based EMC

These naturally formed biomaterials from biological polymers have been used in the manufacture of 3D platforms for breast cancer culture, including Matrigel^®^ [149,150,151], collagen [152,153,154,155,156,157,158,159], HA [160,161,162,163,164,165,166], alginate [1,167,168,169], and gelatin [169,170,171] (Table 2). 

The Matrigel^®^ is the gold standard. It is a tissue formed of a mixture of gelatinous proteins derived from the basement membrane isolated from the mouse sarcoma Engelberth–Holm–Swarm (EHS) and commercially available under the brand name Matrigel^®^ (BD Biosciences) [107,108,109,110] (Table 2). This extract is liquid at 4 °C and turns into a gel at 37 °C under physiological pH and ionic strength. It is then a ready-to-use solution that allows user-defined use. Recently, it has been widely used in 3D experiments in cell biology to assess cell migration, cancer cell behavior, and to create organoids in vitro, as it produces a large amount of ECM rich in type I collagen, laminin-111, heparin sulfate proteoglycans (perlecan) and nidogen [172]. Matrigel^®^ is considered the best product on the market used in the production of most 3D tests performed in cell biology [151,173,174,175,176]. The success of Matrigel^®^ is also due to its biological activity, which allows under normal culture conditions the differentiation of several cell types and the formation of complex structures such as mammary glands of acinous structures [173,177]. In addition, the bioactivity of Matrigel^®^ is due to the presence of soluble growth factors such as fibroblast growth factor (FGF), epidermal growth factor (EGF), transformative growth factor-β (TGF-β), and matrix metalloproteinases (MMP), including MMP-2 and -9. Although easy to use, the presence of these growth factors in unknown and uncontrollable quantities can have an impact on research (positively or negatively) which is why many researchers may prefer to manufacture their hydrogel systems using defined concentrations [24]. In addition, as Matrigel^®^ is produced and purified from an animal, there is a lack of control over its exact composition and batch-to-batch variability in its contents [178]. Similarly, it presents difficulties of handling when it is in a refrigerated liquid state [104]. Although the drawbacks of Matrigel^®^ are significant, Matrigel^®^ is a widely available model for studying many fundamental questions in cell biology, cell adhesion, and cancer research as a versatile platform for in vitro 3D cell culture [151,173,174,175,176]. 

Most natural polymers are structural molecules derived from mammalian ECM. Many different materials were used to develop in vitro 3D breast cancer scaffolding. They were first used as a coating of tissue culture boxes to promote cell adhesion and spread after they are incorporated into 3D materials in different forms (hydrogels, freeze-dried materials, and surface coating of bulk inorganic materials such as elastin, collagens [152,153,154,179], fibronectin, laminin (mainly laminin-111) [152,161,162,179], GAGs (chondroitin sulfate and heparan sulfate, hyaluronan [162,165,166,179], alginate, and gelatin [167,168,169,170,171]. 

Classically, they are purified from ECM-rich animal tissues such as dermis and tendons (collagen type I, elastin), cartilage (collagen type II and GAGs), tumors (laminins and collagen IV), or directly from the blood (fibrin and fibronectin). On the other hand, with progress, it is now possible to obtain some of these molecules from recombinant DNA sources, which makes it possible to work with human ECM molecules, produced with a high degree of purity and free of many pathogens, but they are more expansive [180]. Non-mammalian ECM molecules are also widely used in the design of biomaterials, mainly for their ability to self-assemble in 3D structures, but due to their origin, they lack many aspects of cell adhesion on their structures and should most often be supplemented with adhesive molecules or peptides to obtain a biologically active material. These polymers are hydrogels based on chitin/chitosan (polysaccharide purified from the exoskeleton of fungi or arthropods), agarose, or alginate (both polysaccharides purified from algae), and fibroin (cocoon protein from silkworms and spiders) [181].

- Natural hydrogels

**Figure 1:**
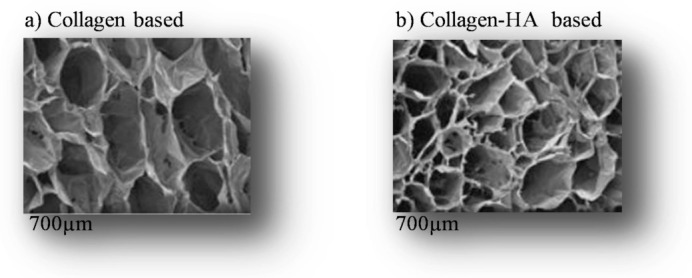
Scanning Electron Microscopy micrographs of the longitudinal sections of freeze-dried scaffolds of (**a**) collagen-based and (**b**) collagen-HA based (adapted from [219]).

Collagen-based hydrogels are natural hydrogels. Collagen, as the main component of ECM, plays a key role in the development and spread of cancer [40,106,182,183,184]. It affects the tumor microenvironment [185,186] especially in breast cancer signaling [182,183], differentiation and migration through cell-matrix interactions [187,188] (Table 2). Depending on the type of tissue, collagen fibrils organize themselves in a variety of ways to form collagen fibers suitable for the specific functions and properties of tissues [189]. The mechanical properties, architecture, and biodegradability of collagen hydrogels can be finely modulated by adjusting their concentrations [190,191] and preparation parameters [136,137,192,193]. Thanks to its specificity of spatial organization and self-assembly of collagen in acid solutions [194], the architecture of hydrogels can be controlled by the manipulation of ionic force, pH, and temperature during frost polymerization [174,195]. The concentration of collagen increases gradually with the regulated evaporation of the solvent, as well as modifies the organization of collagen molecules. The original collagen solution to a solid hydrogel structure (i.e., the so-called “soil/frost transition”) retains the tissue-like molecular organization of collagen molecules [196] (Figure 1a). In addition, an increase in collagen concentration (i.e., the ionic strength of gels) leads to an increase in fiber density and a reduction in pore size but has no effect on fiber diameter [195]. In contrast, increasing temperature and pH accelerates polymerization, reduces fiber diameter and pore size, and also increases the mechanical properties of hydrogel [174,195] (Table 3). As a result, many collagen-based 3D models of in vitro cancer culture have been developed [11,152,153,154,155,158,197,198,199,200,201,202]. 

Polysaccharide-based hydrogels are natural hydrogels. Proteoglycans (PGs) have a protein that is covalently bound to GAG chains. Thanks to the polyanionic profile of GAGs (due to the sulfate groups), they attract water, thus causing the swelling of the GAG [220] (Figure 1b). This swelling can then open pathways of invasion and migration of cells that resemble the state of invasion and cancer metastases [221] (Table 2). These PGs are directly involved in cellular functions and the release of active molecules (growth factors, cytokines) [221]. Hyaluronic acid (HA) is a major category of structural macromolecular components of ECM. It is an unsulfated GAG. The polyanionic nature of GAG attracts water, causing GAG to swell. Concerning HA hydrogel, this natural polysaccharide can be chemically modified to similarly mimic native tumor tissue by adding acrylate or thiol groups that have cross-linked to form a network with a pore size of 70 to 100 nm [220]. Similarly, their sulfation pattern contributes to the binding of growth factors to GAGs [222,223,224]. Indeed, GAGs provide hydration and compressive strength by binding to water as well as are involved in different biological processes such as tumor progression, angiogenesis, and cell development [33,35,39]. It is not only a structural component of the tumor ECM but also a biologically active molecule that has been used extensively in the formation of 3D in vivo tumor models [225]. This use is because of its biodegradable, non-immunogenic, non-inflammatory [205] hydrodynamic and swelling characteristics to fill most of the extracellular interstitial spaces of tissues in the form of hydrated gels [33,35] and promote cancer progression [226] (Table 3). These properties make HA an ideal matrix for preparing 3D tumor models. HA gels are formed by covalent cross-linking (reaction with carboxylic acid groups) with hydrazide derivatives. They carry inherent biological properties such as protein grafting, but they are mechanically poor. HA is most often incorporated into the constituent materials of hydrogels [38,219,227,228,229]. Recent studies have demonstrated the usefulness of HA-based scaffolding for improving adipose tissue development in vivo [230,231] and in vitro [232,233]. Table 4 summarizes the differences between scaffolds made with pure collagen vs. collagen-HA-based ones.

Dextran is a bacterial polysaccharide, consisting mainly of a glucosidic-1,6 bond of D-glucopyranose residues, used for more than 60 years in the medical and biomedical fields [236]. Dextran is widely used for biomedical applications. Its advantages are its biocompatibility, low cost, good water solubility, ease of modification [237], antifouling properties [238,239]. Dextran glucose must be oxidized in duplicate and then followed by a freeze-drying step [240,241,242,243].

Chitosan is the second most abundant natural polymer. It is a linear polysaccharide derived from chitin in the form of a deacetylated derivative [244]. Its advantages are its structure (D-glucosamine bound in (1-4) + N-acetyl-glucosamine) that mimics the structural characteristic of the GAGs of ECM. It’s a biomaterial widely used in biomedical, biocompatible, biodegradable, and can be produced on a large-scale, easily transformed (simple freeze-drying) [245]. It shows a disadvantage of solubility in neutral solutions (it adds cysteine) [246]; no gel is formed without the grafting of cysteine, and poor mechanical properties of the gel are observed [247].

- Synthetic hydrogels

Most synthetic hydrogels are synthesized by polymerization of synthetic polymers, which exhibit versatile biophysical, mechanical, and biological properties in 3D breast cultures to study the relationship between the microenvironment and malignant tumors in vitro [131,133,248]. They are formed of synthetic polymers comprising poly-ε-caprolactone (PCL) [249,250,251], polyethylene glycol oxide (PEG) [38,252,253,254], polyvinyl alcohol or in a mixed solution or a combination of copolymer with poly-lactide-co-glycolide (PLGA) (PLG) [249,250,251,255]. In addition, their use is more preponderant in 3D culture of many cell types including neural [206], bone [207,208], cartilaginous [209,210], muscle [211], and renal cells [212]. Synthetic organic polymers offer a wide range of creativity to produce 3D materials. Although they inherently lack basic biological activity, they have great flexibility in treatment and are easier to be produced [116] (Table 2). The diversity of synthetic polymers used in biomaterials is great as they can be transformed into 3D materials with many types of techniques (electro piping, foaming, hydrogel, and sheets), some of which are not bearable by biological polymers. Polyesters and polyhydroxy acids can be biodegradable thanks to the presence of hydrolyzable bonds in their skeleton, while polyacrylamides and polyacrylates are almost unbreakable in cell culture [117]. Synthetic polymers have the inherent properties to form a 3D scaffolding by which they contain active chemical groups (amine, acid, or alcohol functions) sensitive to chemical reactions, thus providing an ECM model with well-defined characteristics [204,217]. Physiologically, they are irrelevant and can release toxic degradation products to cells [216] (Table 3). Similarly, they are unable to provide the biochemical signals necessary to “communicate” with the cell. To overcome this limitation, synthetic polymers can be functionalized by adding signaling biomolecules, such as peptides, growth factors, and glycans [213,214,215].

Cells embedded in natural biopolymers take advantage of the signaling already present inside the matrix, while synthetic polymers lack signaling patterns capable of modulating the cellular outcome [203]. The stiffness of biomaterials is also very important for cell proliferation and behavior, but an increase in the stiffness of a matrix (PEG gels) acts as a physical barrier for 3D cells, preventing their proliferation and migration [212]. Similarly, studies have already shown that changes in the cross-linking density of PEG-based hydrogel cause changes in cell growth and morphology [218,256,257]. For all these reasons, their applications are limited in the engineering of tumors in vitro [216]. Levenberg et al. (2003) used PLGA and PLA to form porous scaffolding to create a 3D artificial microenvironment for human embryonic stem cell differentiation. While this was partly successful, they also demonstrated the difficulties in getting cells to infiltrate throughout the scaffolding [258]. Similarly, the biodegradation of scaffolding based on poly-lactic acid, poly-glycolic acid, and their copolymer PLGA can lead to the release of by-products such as lactic acid [259,260]. For these reasons, biodegradable materials are not practical for routine 3D cell culture where issues such as storage life, storage, and product consistency need to be taken into consideration [104] (Table 3). 

Nanofibrous scaffolding is a thin sheet composed of synthetic polymer fibers (PLGA or PLGA-PEG) of nanometric size randomly aligned. It forms a fibrous support for ECM and provides topographic features necessary for cell adhesion and growth. These nanofibers can be manufactured by several techniques such as electrospiding, phase separation, and self-assembly with varying chemical properties, diameters, lengths, porosities, and mechanical properties [261,262,263,264,265]. In addition, the authors evaluated the usefulness of this model for drug testing by growing a tumor biopsy on 3D scaffolding and determining the effect of the drug on it. These nanofiber-based models have several key features; for example, providing topographic characteristics to cancer cells for 3D tumoroid development and reproducibility with the ease of tumoroid imaging could make it an elegant approach for drug testing.

**Figure 2:**
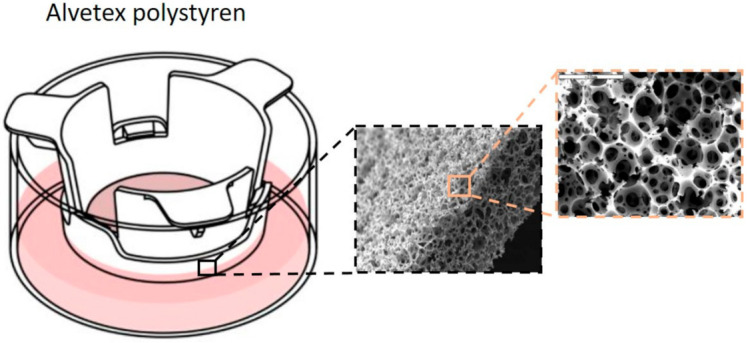
Polystyrene well insert holder for 3D culture Alvetex Scaffold (alvetex^®^/www.interchim.com).

Alvetex^®^ hard-base scaffolding (Figure 2) provides a large internal volume and 3D space that cells can occupy and form a tissue. Cells find a more physiological shape because they are not seeded on a flat surface but in the presence of fibers or sponge-shaped structures with high consistency and reproducibility [119]. These scaffolds are composed of non-degradable inert materials (polystyrene or polycaprolactone PCL) to avoid the formation of by-products. They can be manufactured by electrospinning [120,121,122] or by gas foam technology [123] to improve their application in cell culture, but very few have been developed into a commercially successful process for 3D culture (Table 2). They are important tools in the study of cell-ECM interactions due to the ability of the scaffolding to reproduce the structure of the ECM and high porosity [266,267]. Moreover, it is less affected by cytotoxic compounds. Polystyrene is a familiar substrate to the user in 3D cell culture since it is inert and does not degrade during normal use [55,121,122,268,269] (Table 3). On the other hand, polystyrene has some disadvantages: it is rigid (its rigidity must be controlled), does not have the biomechanical properties found in soft tissues, can display cytotoxicity [270], and a lack of biochemical stimuli (for example, molecules dependent on cell anchorage). However, these can be solved by adding known ECM proteins. In this case, a balance must be found between the needs of the model and the objective of the study [104].

The advantages and disadvantages of the described hydrogel-based scaffolds are summarized in Table 3.

#### 4.1.2. Synthetic Strategies

- Porous material

**Figure 3:**
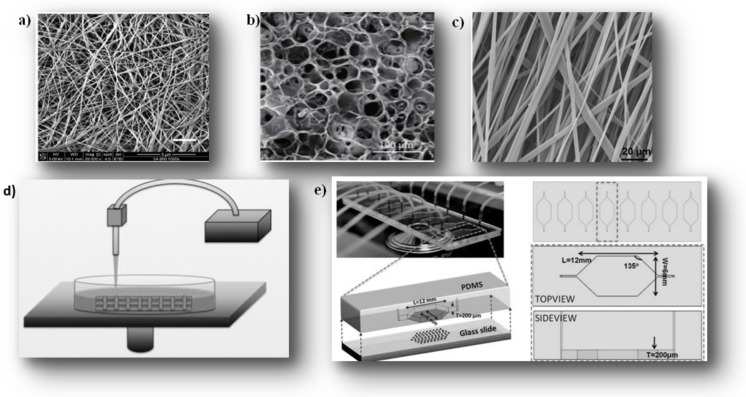
Different synthetic strategies of 3D matrix-based: (**a**) collagen; (**b**) Lyophilization; (**c**) Electrospiding; (**d**) Stereolithography; (**e**) Micro fluid [271,272,273,277,278].

Despite the good biocompatibility of hydrogels due to their water content, they most often have low mechanical properties and high degradation rates. For this, porous materials have been designed with interconnected pore networks and surfaces or fibers to support cell adhesion. These materials are discriminated against 3D models of ECM of nano porous scaffolding where pore structures are in the range of cell diameter (about 10 μm) [130,271,272,273] (Figure 3a). Nevertheless, microporous structures allow more efficient cell penetration and migration into the material but with specific pore sizes since a micro-size can represent a barrier to cellular colonization of the material and limit cellular interactions at the edges of the material [274]. Like hydrogels, porous materials can be prepared with natural and synthetic polymers by different techniques, including electrospinning, phase separation, model creation, and vapor polymerization [274]. The porosity based is mainly used with synthetic polymers (PLA/glycolic acid and PCL) but is also suitable for natural polymers such as collagen and silk fibroin [275]. For example, porous collagen materials, due to their high porous structures, make so-called “collagen sponges”, by thermally induced phase separation. The phase separation of collagen molecules from the water-based solvent is due to the freezing of acidic collagen solution, which induces one that is then eliminated by freeze-drying. Pore size and interconnectivity can be altered by modulating collagen concentration and phase separation temperature or by mixing collagen solutions with other natural polymers such as GAG [276] or synthetic polymers such as PLA. The low mechanical properties of freeze-dried collagen materials are often enhanced by the addition of GAG by cross-linking them with chemical species (aldehydes) using dehydrothermal processes [191].

- Hydrogel technology

The cross-linking of natural hydrogels such as agarose, fibrin, and especially collagen and HA with high water content is one of the popular options in 3D culture that ensures the encapsulation of cells in a hydrogel comprising a loose scaffolding structure [121,279,280,281]. Considerable progress has been made in the preparation of hydrogels called smart hydrogels to better mimic the artificial ECM protein microenvironment [282,283]. However, these hydrogels may undergo unusual changes in the structure, mechanical characteristics, and swelling behavior of the network (support, cell growth), as a result of variations in pH, temperature, light, ionic, and force or electric field or enzymes [284,285,286,287]. Regarding freeze-drying, its main purpose is to regulate porosity and form a collagen sponge. Changing the porosity of hydrogels (the number, size, shape, and interconnectivity of pores) will promote cell growth and homogeneous cell seeding in cell culture [288]. Pore size is also a very important parameter to avoid inhibition of cell penetration. However, the diameters of all cells are less than 200 μm (for nutrition and oxygenation) [289]. To do this, it is necessary to choose the optimal size suitable for each cell type [289,290,291]. A resume of this method, its advantages and disadvantages are summarized in Table 5.

- Collagen Hydrogel by Freeze-Drying (Lyophilization)

Porosity is an amount of open pore volume in a scaffold that provides suitable support for cell colonization, ECM production, and subsequent spheroid formation. Highly porous scaffolding is then essential for the transport of nutrients and the disposal of waste [334]. Pore size (diameter of circular pores or the longest length for non-circular pores) is one of the major factors that affect cellular behavior in the porous matrix, such as migration, adhesion in pores, and interaction with neighboring cells [335]. Lyophilization is a dehydration technique that results in the formation of an interconnected circular/dry oval porous microstructure [276,292,293,294] (Figure 3b). The solution is frozen before undergoing a vacuum drying process leading to the sublimation of ice crystals [234]. This technique is currently the most used for the manufacture of collagen-based scaffolding due to the easy control of the architecture and mechanical properties. In addition, this architecture can be affected by collagen concentration, temperature, and freezing speed [276,295,296]. It has also been shown that a decrease in the final freezing temperature reduces the size of the ice crystal and consequently the size of the pores [297] even for the freezing speed, which must be slow and controlled to result in homogeneous scaffolding (pore shape and size) [298]. Variation in collagen concentration affects the stiffness [234], size and porosity of scaffolding pores since an increase in collagen concentration from 0.5% to 1% (*w*/*v*) increases pore size and reduces scaffold porosity [191,336]. To address the complexity of component alignment observed in the ECM, Campbell et al. used a polycarbonate mold, cylindrical wells, and sharp copper inserts (coated with PTFE). The inserts were thermally insulated from the freeze dryer shelf by a thin 1 mm rubber mat [337,338,339]. In a recent study, Hume et al. used 1% (*w*/*v*) of collagen (bovine Achilles’ tendon) solubilized in 0.05 M acetic acid followed by custom freeze-drying to produce reticulated anisotropic scaffolding (reticulated DAC/NHS). Samples of xenograft mammary tumors and fragment co-culture with 3T3-L1 pre-adipocyte cell line have been successfully cultured in collagen scaffolding (pore size 100 μm), highlighting the promise of ex-vivo application [235]. This is commonly used as a gel for adipose tissue engineering [340,341,342,343], noting that adipocytes increased the migration of tumor cells into scaffolding on day 10 [235]. A resume of this method, its advantages and disadvantages are summarized in Table 5.

- Electrospinning Hydrogel

The electrospinning technique uses electrical forces to form a network of fibers that offer a large surface area from polymer solutions or melts [344] (Figure 3c). This technique is fast, efficient, relatively inexpensive, versatile, and produces microfibers with a diameter of less than 100 nm [345,346]. The diameter of the fibers increases with increasing concentration/viscosity of the polymer [347,348,349,350]. This creates uniform fibers and reduces the incidence of fiber defects such as beading (low concentration or surface tension problems) [348,351]. Fiber thickness generally increases pore size (space between fibers) [299,300], while fibers with a smaller diameter exhibit the opposite effect due to the higher density of the fiber network inside the scaffolding [352]. Noting that manufacturing parameters should be adjusted to avoid overly dense and tight fibrous networks as dense fibrous networks can prevent cellular infiltration into the scaffolding; high porosity is then essential [299,301,302]. In addition, its limited control of porosity and relatively poor mechanical properties reduces its use in a 3D culture based on hydrogel scaffolding [303,304,305,306] (Table 5).

Collagen-based scaffolding manufactured by electrospiding uses 1,1,1,3,3,3-hexafluoroisopropanol (HFIP) [307,353], trifluoroethanol (TFE) [308,309] solvents, although these nanofibrous scaffolds were wrung out using a more benign water/salt/alcohol solvent system [310]. The diameter of the fibers in these collagen-based scaffolds ranged from 100 to 900 nm, with differences obtained by changing the specific electrospiding parameters. On the other hand, crosslinking agents can be used to increase the mechanical properties of electrospirated scaffolding [307,309,310,311,312,353]. Highlighting the potential of collagen-based scaffolding in 3D in vitro culture model, excellent cell proliferation and viability has been observed in many manufactured models [307,311,312,354]. Szot et al. used this model to assess cellular behavior on electrospirated collagen/PCL fibers manufactured in terms of growth, proliferation, adhesion, and infiltration (fiber diameters ~400 nm (5%) to 2250 μm (15%) depending on concentration) [299]. Recently created (2021) by Malakpour-Permlid et al., a 3D culture model is based on a PCL fiber network [355] resembling the collagen network of ECM due to its involvement in the main tissues and organs of the body. And as collagen is one of the most widely used biopolymers in tissue engineering [81,356], this fiber network is applied in the culture of normal and cancer cells by mimicking the collagen structure that ensures the 3D fixation and growth of cells. Despite the advantageous bio-activity in vitro and in vivo of collagen-based hydrogels and biomaterials, the major problem of clinical translation and therapeutic use remains related to the animal origin of collagen (i.e., bovine collagen, of porcine origin type I), with potential pathogenic content (disease transmission) [357] and lack of growth factor (addition of TGF-B) [355].

- 3D-Printing Scaffolding for 3D cell Culture via Stereolithography

Stereolithography is another method commonly used for imitation of complex structures in vitro of the intestine artificial 3DP models such as microvilli. It is an excellent candidate for studying homeostasis regeneration mechanisms in vitro. It is based on a construction of the different layers that harden to visible light or infrared [313,314] (Figure 3d). Each layer will be superimposed after the next layer has dried (generally by UV). These layers are printed by a specific thin sheet material: The Installation Multiresolution 3D Printer (Dilase 3D, Kloe France), layer by layer until the scaffolding is finished [313]; it is then placed under UV light where it is post-cured [315,316,317]. This support can be combined with a polymerizable photo hydrogel (PCL fibers, PCL/gelatin) that promotes cell line growth with 3D-printing stereolithography and produce different scaffolds size from mm to cm. However, the manufactured scaffolding is usually limited to a few tens of microns of resolution and needs specific end expensive equipment [277,316,317,318,319,358] (Table 5).

- Micro Fluid

The microfluid support consists of silicon/elastomer-based devices. It creates a dynamic microenvironment presenting micro-channels with proportions from 1 to 1000 μm which are responsible for the exploitation of a small volume of fluids generally in the range of 10–9 to 10–18 (Figure 3e). In this system, the flow of the fluid is strictly laminar (in parallel layers) rather than turbulent (parallel and no strong) [320,321]. This support is normally applied in complex 3D structures or to synthesize matrices used in human transplantation. The generation of aggregates of different forms and the coculture of several cells reconstitute a more physiologically relevant tumor [322,323]. It simulates cell-cell contacts and biological signals controlled by spatial and temporal gradients of soluble biological factors [324,325,326], progression, invasion, angiogenesis as well as treatment efficacy [320,327] since the continuous flow of nutrients and therapeutic agents establishes a physiological profile such as that of blood circulation and intravenous injections. In fact, this system saves labor and the reagents used since it works automatically and consumes low cellular usage [278,328]. According to its manufacturing complexity, this system requires professional equipment and special design [322,323] and a higher budget than other strategies applied [329,330,331,332,333]. The different synthetic strategies are summarized in Table 5.

### 4.2. Scaffold-Free Spheroids

Self-assembly is a natural phenomenon that occurs during morphogenesis and organogenesis. In culture, these techniques are considered the least complicated to apply because of the absence of a fastening surface or scaffolding that allows cell colonies to self-assemble and form aggregates of non-adherent 3D microtissues called spheroids [359,360,361,362,363,364,365,366]. According to a general definition, spheroids are aggregates of cells growing in a 3D way in suspension; they can be mono or multicellular (homo or heterotypic). The cells form hard spherical structures with a well-balanced morphology of variable size (50 to 150 μm), formed by a necrotic nucleus and a peripheral layer [10,367,368,369]. Historically, Holt Freter was the first to use spheroids as a morphogenic model in his investigation of skin behavior during development in 1944 [370]. Then this mode was considered a powerful tool in research and clinical applications and is the best in vitro cellular model for high-throughput screening [138,371,372,373]. Recent advances in tissue engineering and regeneration have provided new techniques for the generation of tumor 3D spheroids for a variety of cancer types in vitro. This model of multicellular tumor spheroid was initially created in the early 1970s by radiobiologists, and then it was managed to be used for a wide variety of cancer cell lines. Nowadays, a multitude of techniques are used for the production of spheroids because cells are unable to adhere to the support [7,363,364,365,374,375], either in the adhesion-free gel of the micro-well with superposition of cellular suspensions (e.g., agarose gel or alginate gel) [376,377,378,379,380,381] or by other techniques as pellet culture, suspended goutte and filature culture [382,383,384].

Spheroids have been widely used by a simple, high-yield, inexpensive application protocol that allows the production of more spheroids to mimic the architectural and functional characteristics of native tissues [138,371,372,373,385,386], and to assemble models of different types of cancer in vitro such as breast cancer (spherical shape of the breast canal) [363,380,381,387]. On the other hand, this culture requires intense and precise work and presents a risk of the formation of spheroids of uniform size and shape [376,377,378,379,380,381]. Noting that this variation may be at the origin of the gel used but, in general, the gels (agarose, alginate, chitosan) used in the spheroid formations have benign characteristics such as plant origin that do not present a risk of animal contamination, significant stability at room temperature (but are biodegradable) and non-toxicity [167,168,169,369,388]. In contrast, agarose gels were formed by heating (near the boiling temperature) the solution that freezes with cooling. For these reasons, different porous architectures and mechanical properties can be constructed according to the modulation of agarose concentration [38]. For alginate citing a commercial product AlgiMatrix™ ready to use, it looks like a highly porous sponge (>90%), ready to use (freeze-dried alginate), stable at room temperature with long-term viability, non-toxic, biodegradable, and can easily be degraded by a dissolution buffer in a few minutes leaving cell aggregates intact for analysis (spheroids 50 to 150 μm) [10,367,368,369]. Similarly for chitosan (derived from crustaceans) has no binding domain to human cells therefore favorable to spheroids [245,377,389,390]. Table 6 summarizes the advantages and disadvantages of spheroid culture.

#### 4.2.1. Technical Methods of Spheroid Formation

- Pellet Culture

**Figure 4:**
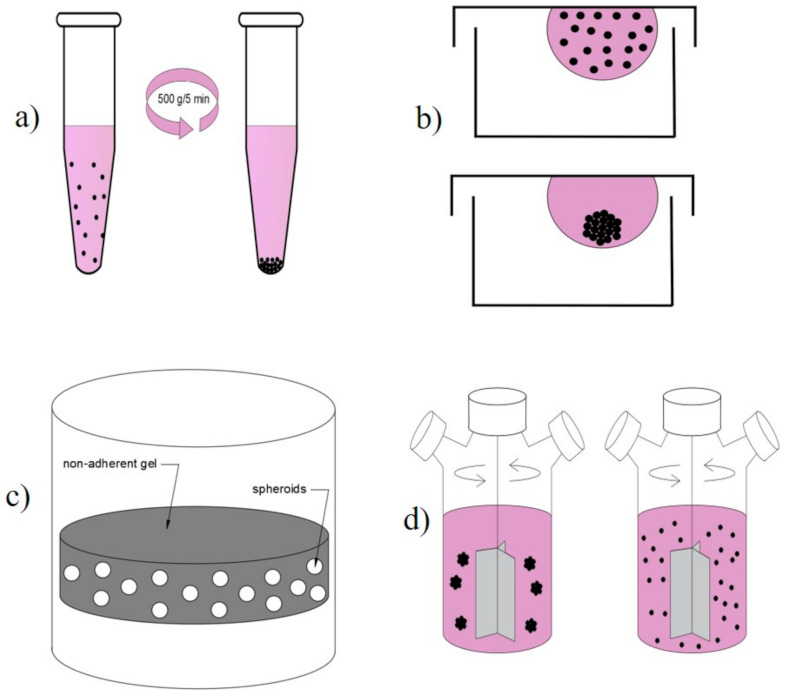
Technical methods of spheroid formation: (**a**) Pellet culture; (**b**) Hanging drop; (**c**) Liquid overlay; (**d**) Spinner culture.

In this system, cells are concentrated at the conical bottom of a tube by centrifugal force (500 g, 5 min) to maximize cell-to-cell adhesions [359,391] (Figure 4a). After that, the supernatants are removed, and cell pellets are resuspended in a spheroid formation cell culture medium. We noted that shear stress due to centrifugation could damage cells, so to optimize results, the suspension can be incubated on an agitator for one hour before centrifugation [361,392,393,394,395,396] (Table 7).

- Hanging drop

Hanging drop is a spheroid culture technique that uses surface tension and gravitational force to form definite size spheroids in the form of droplets that rely on gravity self-disassembly. It allows single cells to aggregate and fabricate spheroids in the form of droplets (Figure 4b) [300,301]. By controlling the volume of the drop or density of cell suspension, it is possible to control the spheroid size. We prepare a cell suspension at desired density distribution in the wells of a mini-plateau, then it will be placed on the mini-tray, and the entire mini-tray is overturned upside down [397,398]. The drop remains fixed on the mini tray on the inverted surface (surface tension). Therefore, this technique is mostly used due to the defined and controlled size of the spheroid (drop volume and suspension density) with inexpensive equipment. Despite high amount production (384 spheroids in a single network), heterotypic spheroids can appear with narrow size distribution [359,361,383,397,398,399,400] (Table 7).

- Cultivation of Molded Lozenges and Liquid Overlay (Static Suspension)

The cultivation of molded lozenges and liquid overlay (static suspension) are culture technique that forms spheroids by interrupting the adhesion of cells on non-adherent culture plates or gel with non-adherent properties such as agarose with micro-well (agar) gel or pHEMA with superposition of cellular suspensions (Figure 4c) [359,361,401,402,403,404]. It is a simple method to monitor the formation and growth of spheroids. Since the cell binding to the support is inhibited, cells spontaneously form spheroids. It is forced to aggregate by continuous agitation with/without centrifugation. Despite the excellent non-adherent properties of agarose, this biomaterial has drawbacks in terms of producing heterogeneous spheroids in size and shape (Table 7) [359,361,376,399,403,404,405,406].

- Spinner Culture Technique

It refers to the technique wherein the cell suspension in spinner centrifugal flask bioreactor, generated by a magnetic stirring wheel container, and which is continuously mixed by convection force stirring (Figure 4d). It adds the uniform and well-mixed single-celled suspension with constant continuous stirring to form the spheroid (may not be useful for cells with low cohesion; they have the risk of apoptosis). The stirring rate must be constant because a high stirring rate induces damage to the spheroid cells and a slow speed makes the cells sink to the bottom of the container (blocks the spheroids). In addition, it is difficult to follow the spheroids during formation (Table 7) [26,359,361,407,408,409,410,411]. 

Table 7 summarizes the technical methods of spheroid formation.

#### 4.2.2. Technical Methods of Tumor Spheroid Formation

Due to their particular interest, spheroids are the most applied 3D models in oncogenic research. They form an effective tool capable of studying the variation in morphology, topography, size, cell organization, protein expression, and genes in the invasive and metastatic potential of cancer cells [363,380,381,382,383,384,387]. These tumor spheroids have a heterogeneous distribution with active cells proliferating on the surface of spheroid cells (oxygen and nutrients) and resting cells in the center [104,366]. Tumor spheroids may be homotypic formed only of cancer cells or heterotypic consisting of cancer cells with other cell types [375]. Spherical cancers can be classified into four groups:

- Multicellular tumor spheroids are obtained after aggregation and compaction of the cultured cell suspension (1–7 days) under non-adherent conditions (well plates, vials or boxes + agar gel, agarose or polyH + a traditional culture medium depending on the cell line) [399,412,413].
- Tumorospheres (floating sphere): Tumors are formed from a single cell capable of giving rise to a sphere by clonal expansion (5–7 days up to 1–2 months) under conditions of low adhesion (plastic with low adhesion) and with a stem cell medium (depending on the type of cancer, growth factors may be preferentially added) [414,415,416,417,418].
- Tissue-derived tumor spheres (endoscopic biopsy): Tumor spheres derived from cut (scalpel blade) and minced partially dissociated cancerous tissues are generated by partial dissociation of tumor tissue and compaction/remodeling (2–5 days up to 12–18 days) in conventional FBS-supplemented medium [416,419,420].
- Organotypic multicellular spheroids are formed from the cutting of partially dissociated tumor tissue (mechanically or enzymatically) under non-adherent conditions (plastic treated in culture and then non-adherent conditions) that have rounded during culture (1–3 days) [419,421,422,423].

## 5. Conclusions

Standard cell culture studies are widely used to delineate biological, chemical, and molecular pathways, first by traditional 2D culture and then by enhanced 3D culture. According to 2D cell limitations in some practices, recent advances in tissue engineering and regeneration then provided new techniques for a variety of 3D in vitro models. Cells develop in an organized three-dimensional (3D) matrix, and their behavior depends on interactions with immediate neighbors and ECM. The 3D culture can provide an important tool for better understanding changes, interactions, and cellular and molecular signaling during malignant transformation and metastasis. Three-dimensional (3D) cellular scaffolding is then essential for tissue engineering. So far, various natural and synthetic polymer hydrogels have been used to design 3D scaffolding as biomaterials. This is a barrier to mimicking the native ECM microenvironment, and therefore synthetic scaffolds may be more useful for investigations of specific tumorigenic steps. We provide here characteristics, advantages, and disadvantages of 3D cell culture compared to 2D types, different types of 3D matrices such as natural, synthetics hydrogel, and spheroids—the best rational classification of the most used 3D strategies models in cancer research. Finally, depending on the specific objectives, the most relevant 3D models must be carefully selected.

## Figures and Tables

**Table 1:** Comparison between 2D and 3D culture models.

Characteristic	2D	3D	References
**Support for cell fixation**	Utensils (plastic, polycarbonate)	Extracellular matrix in vitro	[2]
**Instructions for use**	Traditional culture	Imitating the natural microenvironment	[11,12,13,25,26,52]
**Interaction and communication**	Cell-cell (co-culture)	Cell-cell and cell-matrix 3D interactions	[53]
**Cell forms**	Flat and extensible	Natural cellular structure preserved	[23,24,43,45]
**Media cell interface**	Homogeneous exposure of all cells to the media	Heterogeneous exposure (the upper layer is more exposed than the lower layer)	[26]
**Cell junctions**	Less common	More common (cell-cell communication)	[3,10,12,54]
**Cell differentiation**	Moderately and poorly differentiated	Well-differentiated	[43,44]
**Cell proliferation**	Higher proliferation rate than in the natural environment	Medium or high proliferation rate depending on cell type and 3D culture technique	[45,50,51]
**Treatment sensitivity**	Cells more sensitive to treatment	Cells less sensitive to treatment	[46,47]
**Viability**	Sensitive to cytotoxins	High viability and less sensitivity to external factors	[55]
**Cost**	Cheap	Expensive	[7,8]

**Table 2:** Scaffold-based 3D techniques overview with attribute.

Technique	Protein-Based EMC	Natural Hydrogels	Synthetic Hydrogels	Hard Polymer Scaffold
Product description	Matrigel^®^	Collagen, hyaluronic acid	TrueGel3D (polymers with crosslinkers)	Polystyrene-polycaprolactone Alvetex
Biological relevance	Effective +++	Effective +++	+/−	+/−
Consistency/reproducibility	Low −	High ++	Very high+++	Very high +++
Risk of contamination	Low −	High++	Very high+++	Very high +++
Modularity/customization	Low −	Moderate +	High ++	low −
Cell recovery	+/−	+	++	+++
Downstream analysis (imaging, molecular analysis)	+	++	++	++
References	[107,108,109,110]	[111,112,113,114,115]	[116,117,118]	[119,120,121,122,123]

**Table 3:** Advantages and disadvantages of hydrogel-based.

Hydrogel	Advantage	Disadvantages
Matrigel^®^	-Widely available -Frequently used in cancer research [151,173,174,175,176]	-Unknown and uncontrollable amount growth factors [24] -Lack of control over its exact composition -Variable from batch to batch [178] -Difficulties of handling when it is in a refrigerated liquid state [104]
Based on Collagen	-Good adhesion and cell migration support [145,146,147,148,156] -Biocompatibility, mechanical strength, degradability, and limited immunogenicity [157,158] -The most widely used tissue engineering and in tumor culture [11,152,158,199,200,201,202] -Cell signaling patterns [140,141,142,143,144,203]	-Animal origin can potentially transmit pathogens [204] -Biodegradable [159]
Hyaluronic acid	-Provide hydration and resistance for cellular mechanisms [33,35,39] -Biodegradable, non-immunogenic, non-inflammatory [205] -Hydrodynamic and swelling [33,35]	-Animal origin can potentially transmit pathogens[204]. -Mechanically poor [38] -Biodegradable [159]
Synthetic (PEG), (PCL), (PLA) (PGA)	-Most used in 3D neural culture, bones, cartilaginous, tissue, and kidney tissue [206,207,208,209,210,211,212] -A defined chemical composition and adjustable mechanical properties for cultivation [213,214,215] -Available [119] -Easily modified and formulated with different rigidity only the type of fabric [119]	-Physiologically irrelevant and may release toxic degradation products to cells [216] -Limited applications in in vitro tumor engineering [216] -Contains active chemical groups sensitive to chemical reactions [217] -Irrelevant and may release toxic degradation products to cells [216] -Biophysical parameters (mechanical properties and permeability, stiffness) must be considered [119,206,212] -Loss of cell signaling patterns [203] -Sensitive to pH (PEG) [218]

**Table 4:** Comparison of technical characteristics between scaffolds made with pure collagen vs. collagen-HA-based ones [219,227,228,229,234,235].

	Pure Collagen	Collagen-HA
Technique	By lyophilization 1%	By lyophilization 1%
Pore size	100 et 220 μm	100 et 220 μm
Porosity	Similar	Similar
Denaturation	Absent	Absent
Efficacity	++	+++
Resistance of dissolution	+	++
Dissolution hydrolyte	19.2% in 7 days	11.4% to 13.3% in 7 days
Cellular proliferation	++	+++

**Table 5:** Comparison of different synthetic strategies of 3D matrix-based.

Fabrication Method	Method Overview	Scaffolding Morphology	Advantages	Disadvantages
Hydrogels [11,54,193,279,280,281]	-Collagen gel solution (usually type 1 collagen and acetic acid) mixed on ice and usually neutralized (NaOH) and then gelled -Physical parameters: collagen, pH, the temperature of desired gelling	-Dense gel network of string-like fibers. The thickness of the fiber depends on the manufacturing parameters	-Easy to apply -Matrigel is widely used in cancer research, so many user guides are available -High level of cell viability	-The least porous -Risk of poor distribution of cells and nutrients. -An architecture is more difficult to control, therefore, has less reproducibility of the exact architectures desired -Poor mechanical properties before cross-linking
Lyophilization [153,276,292,293,294,295,296,297,298]	-Creation of a homogeneous suspension of collagen with acid (usually acetic acid) at high speed -Heat treatment (controlled or quenched) for the sublimation of ice crystals under vacuum to the defined freezing point before returning to ~0 °C The dried scaffolding must reach room temperature to complete the process	-Interconnected network -Highly porous -A well-defined pore shape and sizes	-Good control of scaffolding architecture -A wide production range in terms of pore sizes and orientation -High porosity levels. -Inexpensive-High level of cell viability	-Problems in the freezing process affect the final scaffolding architecture from one batch to another -Poor mechanical properties before cross-linking
Electrospiding [299,300,301,302,303,304,305,306,307,308,309,310,311,312]	-Collagen solubilized (usually HFIP or TFE) and added to the syringe/injection system -A high-voltage electric field is applied, causing the charge of the solution, the eruption of the polymer fiber of the tip of the needle, and the whip of the liquid jet -The solvent evaporates during the process, leaving a network of dried fibers deposited on the collection plate (non-woven or aligned)	-Dense and tight fiber array (chain-shaped) of nanometric or micro size	-Fibrous network that closely resembles native collagen fibers. -Wide range of size/diameter/achievable fiber pattern -High level of reported cell viability	-Use of harmful solvents (collagen scaffolding) -Solvents are expensive -Dense fiber networks can reduce the level of cellular infiltration.
Stereolithography [277,313,314,315,316,317,318,319]	-prints layer by layer a UV-curable material in thin sheets -Installation of a multiresolution 3D printer (Dilase 3D, Kloe France) -Each layer is superimposed after drying the next layer -Use of different light sources (visible, UV, IR) capable of polymerizing photosensitive materials.	-Hard layer set (UV)	-Capable of producing scaffolding of size mm to cm -Can be combined with different components to hydrogels or electro spinning (PCL fibers, PCL /gelatin) -high differentiation rates and adhesion -Imitates complex structures in vitro: as villi of the intestine	-Specific equipment -Expensive -Manufactured scaffolding is usually limited to a few tens of microns of resolution
Micro fluid [278,320,321,322,323,324,325,326,327,328,329,330,331,332,333]	Support consisting of silicon/elastomer-based devices having microchannels with proportions from 1 to 1000 μm that exploit a small volume of fluids (10-9 to 10-18 L). These fluids are continuous flows of nutrients and therapeutic agents, establish a physiological profile such as that of blood circulation and intravenous injections	-Matrix that has micro channels- which can be either strictly laminar (in parallel layers) or turbulent (parallel and strong numbers)	-Labor-saving -Microenvironment dynamics (fluid flow) -Generate aggregates of different forms Co-culture of several cells -Simulates cell-cell contacts and biological signals controlled by spatial and temporal gradients of soluble biological factors -Study tumor progression, invasion, angiogenesis as well as treatment tests -Low reagent consumption and low cell utilization	-Requiring professional equipment and special design -Complexity. -High cost

**Table 6:** Advantages and disadvantages of spheroid culture.

Advantage [371,372,373,376,377,378,379,380,381]	Disadvantage [359,366,376,377,378,379,380,381]
Inexpensive High efficiency Improves cell viability and proliferation Retains intrinsic phenotypic property Keeps physical interactions that more closely reflect behavior in the three-dimensional native tissue (3D)	Variable diameter and size Intense work Diffusion gradient depends on the size (oxygen nutrient, paracrine factor) that decreases inwards Self-disassembly is affected by the rate of production and consumption of factors

**Table 7:** Technical methods of spheroid formation.

Technical Methods	Means of Application	Mode of Operation	Advantages and Disadvantages	References
Pellet Culture	Concentrate the cells at the conical bottom of a tube by centrifugal force (500 g/5 min)	-Remove the supernatants to collect the cell cap -Capus resuspended in a culture medium to form the spheroids -To optimize: the suspension can be incubated on an agitator for one hour before centrifugation	-Maximized cell-to-cell adhesions -Suitable for the differentiation of mesenchymal cells, chondrogenesis, and bone formation -Disadvantage: Shear stress due to centrifugation can damage cells	[359,361,391,392,393,394,395,396]
Hanging drop	Use of surface tension and gravitational force to form spheroids in the form of droplets that rely on gravity self-disassembly	-Preparation of a cell suspension at desired density distribution in the wells of a mini-plateau -Placed a lid on the mini-tray, and the entire mini-tray is overturned upside down -The drop remains fixed on the mini-tray on the inverted surface (surface tension)	-Most commonly used -Defined and controlled size of the spheroid (drop volume and suspension density) -Coefficient of variation narrow size distribution from 10 to 15% -Inexpensive equipment -A large amount can be produced-Heterotypic spheroids (’to 384 spheroids in a single network)	[359,361,383,397,398,399,400]
The cultivation of molded lozenges	Non-adhesive gel (agarose) usually prepared in molds	-Cells are forced to aggregate by continuous agitation -Can be accelerated by centrifugation	-Removes restrictions on spheroid size -Increases production rate -High centrifugation can disrupt spheroids (function)	[361,401,402]
Liquid overlay (static suspension	Materials that do not adhere to cells that inhibit cell attachment, such as agarose (agar) gel or pHEMA	Cell bindings to the support are inhibited; cells spontaneously form spheroids	-Coefficient of variation narrow size distribution from 40% to 60% -Easy to monitor the formation and growth of spheroids in a plate 96 wells -Simple Method -Heterogeneous spheroids in size and shape	[359,361,376,399,403,404,405,406]
Spinner Culture	Use of convection force by stirring the bar in centrifugal flask bioreactor containers generated by a magnetic stirring wheel or bar	Add the uniform and well-mixed single-celled suspension with constant continuous stirring	-The spheroid depends on the size of the bioreactor container -Speed must be constant -A high stirring speed affects the spheroids and a slow speed makes the cells sink to the bottom of the container (blocks the spheroids) -Forms heterotypic spheroids -May not be useful for cells with low cohesion (risk of apoptosis) -it is difficult to follow the spheroids during formation	[26,359,361,407,408,409,410,411]

## Data Availability

All pertinent data are presented within this manuscript.

## Abbreviations

2D: two-dimensional; 3D: three-dimensional; ECM: extracellular matrix; GAG: glycosaminoglycan; HA: hyaluronic acid; MMP: metalloproteinases; PCL: poly-ε-caprolactone; PEG: polyethylene glycol; PG: proteoglycans; PLA: poly-l-lactic acid; PLGA: lactic-co-glycolic acid.
